# An Integrated Approach to the Anti-Inflammatory, Antioxidant, and Genotoxic Potential of Portuguese Traditional Preparations from the Bark of *Anacardium occidentale* L.

**DOI:** 10.3390/plants13030420

**Published:** 2024-01-31

**Authors:** Sofia Encarnação, Katelene Lima, Quintino Malú, Gonçalo I. Caldeira, Maria Paula Duarte, João Rocha, Beatriz Silva Lima, Olga Silva

**Affiliations:** 1 Research Institute for Medicines (iMed.ULisboa), Faculty of Pharmacy, Universidade de Lisboa, 1649-003 Lisbon, Portugal; sofia.encarnacao@edu.ulisboa.pt (S.E.); k.lima@edu.ulisboa.pt (K.L.); quintinomalu@office365.ulisboa.pt (Q.M.); goncalo.caldeira@edu.ulisboa.pt (G.I.C.); joao.rocha@edu.ulisboa.pt (J.R.); mblima@ff.ulisboa.pt (B.S.L.); 2 MEtRICs/NOVA School of Science and Technology, Universidade NOVA de Lisboa, Campus de Caparica, 2829-516 Almada, Portugal; mpcd@fct.unl.pt

**Keywords:** cashew, ethnopharmacology, free radical scavenger, inflammation, pre-clinical safety, phytochemistry

## Abstract

*Anacardium occidentale* L. stem bark Traditional Herbal Preparations (AoBTHPs) are widely used in traditional medicine to treat inflammatory conditions, such as diabetes. The present study aims to evaluate the anti-inflammatory, antioxidant, and genotoxic potential of red and white Portuguese AoBTHPs. Using a carrageenan-induced rat paw edema model, a significant anti-edema effect was observed for all tested doses of white AoBTHP (40.2, 71.5, and 127.0 mg/kg) and the two highest doses of red AoB THP (71.5 and 127.0 mg/kg). The anti-edema effect of red AoBTHP’s highest dose was much more effective than indomethacin 10 mg/kg, Trolox 30 mg/kg, and Tempol 30 mg/kg. In DPPH, FRAP, and TAC using the phosphomolybdenum method, both types of AoBTHPs showed similar antioxidant activity and no genotoxicity up to 5000 µg/plate in the Ames test. The LC-UV/DAD-ESI/MS fingerprint allowed the identification of gallic and protocatechuic acids as the two main marker compounds and the presence of catechin, epicatechin, epigallocatechin gallate, and ellagic acid in both AoBTHPs. The obtained results support the validation of red and white AoB and their THPs as anti-inflammatory agents and contribute to the possible development of promising new therapeutic options to treat inflammatory conditions.

## 1. Introduction

Inflammation is a physiological response of the immune system to tissue injury, foreign organisms, toxic compounds, or radiation exposure [1]. In these cases, inflammatory responses can be induced to remove noxious stimuli and initiate the healing process by activating immune cells and inflammatory signaling pathways [2,3]. Clinically, acute inflammation is characterized by pain, heat, redness, swelling, and loss of function [4]. If acute inflammatory responses fail to restore tissue homeostasis, the inflammation can become chronic and lead to significant destruction of the injured tissue [5,6]. Chronic low-grade inflammation, characterized by persistently elevated levels of circulating pro-inflammatory cytokines, contributes to, or at least exacerbates, a wide range of debilitating non-communicable diseases, such as cancer, cardiovascular disease, and diabetes mellitus, making a significant contribution to the global disease burden [3,7].

Reactive oxygen species (ROS) generated from various biological (e.g., normal cell functions) and environmental sources (e.g., ultraviolet radiation) may directly or indirectly mediate the inflammation and tissue dysfunction associated with inflammatory conditions [8]. Furthermore, excessive production of ROS and reduced antioxidant concentration can promote oxidative stress [9]. It is accepted that post-inflammatory oxidative stress can potentiate the inflammatory response through different pathways and vice versa [10].

Pharmacological treatment of inflammatory diseases usually includes non-steroidal anti-inflammatory drugs [11]. They promote the suppression of prostaglandin biosynthesis by inhibiting the enzyme cyclooxygenase (COX). However, there are relevant gastrointestinal and cardiovascular systemic adverse effects associated with prolonged use of such drugs, respectively, due to the inhibition of COX-1 and COX-2 enzymes [12]. For these reasons, the pursuit of new, safer, and effective anti-inflammatory agents is needed and includes the study of medicinal plants used in traditional medicine as a source of anti-inflammatory traditional herbal preparations and natural products [13,14].

The practice of traditional medicine combined with the ethnobotanical knowledge of populations has led to numerous reports on the use of medicinal plants in pathologies with an inflammatory concern [15,16,17]. Based on these reports, laboratory studies have developed in recent decades, focusing on scientific validation of the traditional uses of these plants and on the discovery of novel drugs [18,19].

One of the most popular species native to South America is *Anacardium occidentale* L., commonly known as the cashew, which has been widely used in traditional medicine to treat various diseases [20,21,22]. The use of *Anacardium occidentale* bark (AoB) in traditional medicine to treat inflammatory conditions has been reported in different African and American countries [20,23,24]. Therefore, several pre-clinical studies have been conducted to enhance the knowledge about the anti-inflammatory effect of AoB extracts. Olajide et al. (2013), using lipopolysaccharide-stimulated microglia, found that the anti-inflammatory properties of a methanolic AoB extract were associated with the inhibition of inflammation-associated cytokine production and inducible nitric oxide synthase and COX-2 gene expression by blocking nuclear factor kappa-light-chain-enhancer of activated B cells (NF-κB) and mitogen-activated protein kinase (MAPK) signaling pathways in microglia [25]. Despite the different modes of preparing the AoB extracts (solvents, medicinal plant-to-solvent ratio, and type of extraction) and the selected models, all in vivo studies showed the anti-inflammatory activity of the AoB-tested samples (Table 1) [26,27,28,29,30].

Portuguese traditional herbal preparations (THPs) based on aqueous extracts of red and white types of *Anacardium occidentale* stem bark (AoB) have been used in the Community of Portuguese Language Countries for more than 30 years to control type 2 diabetes by oral administration [23], a relevant inflammatory condition [31,32]. 

In our laboratory, these AoBTHPs have already undergone phytochemical and pharmacological evaluations to scientifically validate their traditional use in diabetes treatment and to ensure their safety [23,33,34]. In a 14-day repeat-dose toxicity test in mice, no treatment-related signs of toxicity were seen at doses up to 402 mg/kg of the red and white AoBTHPs. The micronucleus test and comet assay performed on CD-1 mice administered a single dose of 2000 mg/kg (*per os*) of each AoBTHP showed no in vivo genotoxic potential [23]. Both AoBTHPs proved to be a source of natural antioxidants with free radical scavenging potential in the 1,1-diphenyl-2-picrylhydrazyl (DPPH) assay [23,35]. The hypoglycemic activity was observed at doses of 40.2, 71.5, and 127.0 mg/kg/day of red AoBTHP using a db/db mouse model. The highest AoBTHP dose showed a more potent antidiabetic effect than glibenclamide [34]. The botanical identification parameters have been established for the red and white AoBs as raw herbal materials for pharmaceutical use. In addition, the chemical profiles of their marker compounds were established using thin-layer chromatography and high-performance liquid chromatography coupled with an ultraviolet photodiode array detector (LC-UV/DAD). Gallic and protocatechuic acids were identified in both AoB types [33,34,35]. Furthermore, spectrophotometry has been used to quantify secondary metabolites on AoB, with the results showing that condensed tannins and triterpenoids are the main classes of secondary metabolites [23,33].

Our aim is to enhance the knowledge of the therapeutic potential of the aforementioned Portuguese traditional formulations made from raw materials subjected to pharmacognostic characterization, which have already been evaluated in vivo for their antidiabetic potential and preclinical safety. Given the correlation between the mechanisms of inflammation and diabetes, this work includes the evaluation of the in vivo anti-inflammatory and in vitro antioxidant activity of these preparations using widely accepted and well-established methodologies, namely the anti-inflammatory activity through carrageenan-induced rat paw edema model and in vitro antioxidant activity using DPPH method, Ferric Reducing Antioxidant Power (FRAP) assay, and Total Antioxidant Capacity (TAC) by the phosphomolybdenum method. Additionally, phytochemical profiles of red and white AoBTHPs will be characterized, and their marker compounds will be identified by Liquid Chromatography-Ultraviolet/Diode Array Detector-Electrospray Ionization-Tandem Mass Spectrometry (LC-UV/DAD-ESI-MS/MS), as well as the assessment of in vitro genotoxic potential by the Ames test.

Through this approach, we intend to advance our understanding of the therapeutic potential of these chemically characterized red and white Portuguese AoBTHPs at specific tested doses to treat inflammatory diseases.

## 2. Results

### 2.1. Genuine Drug Extract Ratio and Extract Chemical Standardization

The genuine drug extract ratio (DER), i.e., the ratio of the amount of starting herbal substance to genuine herbal preparation [36], was 1:7.62 for red AoBTHP and 1:7.92 for white AoBTHP. The standardization of Portuguese AoBTHPs was previously determined based on their total phenolic content using the Folin–Ciocalteu method, and the obtained values were 31.39 ± 0.50 mg GAE/g AoB and 31.36 ± 0.54 mg GAE/g AoB, for red and white AoBTHPs, respectively.

### 2.2. LC-UV/DAD-ESI-MS/MS Phytochemical Profile

**Figure 1:**
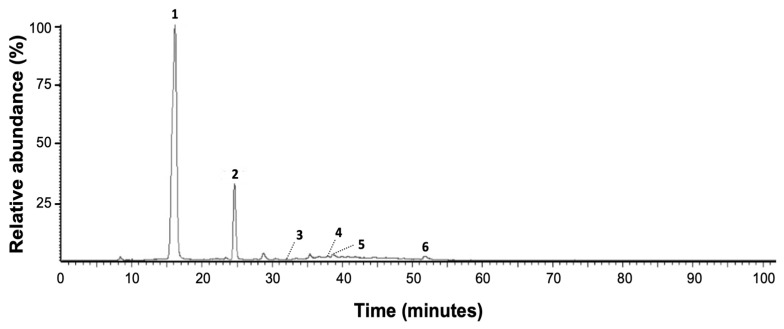
Red AoBTHP LC-UV/DAD Maxplot wavelength (*λ* = 210–600 nm) chromatogram profile. 1: gallic acid; 2: protocatechuic acid; 3: catechin; 4: epicatechin; 5: epigallocatechin gallate; 6: ellagic acid.

The typical chromatographic fingerprint of red AoBTHP is shown in Figure 1. The chemical profile of white AoBTHP is similar to that of red AoBTHP.

The results clearly show the presence of six peaks (peaks 1–6). Data on the chromatographic properties of these peaks are shown in Table 2. Retention time, UV/DAD absorption spectrum, and mass fragmentation patterns in negative ion mode are given. Based on these chromatographic and spectral data, the presence of gallic acid (peak 1), protocatechuic acid (peak 2), catechin (peak 3), epicatechin (peak 4), epigallocatechin gallate (peak 5), and ellagic acid (peak 6) is determined by comparison the data obtained with the PubChem database [37], literature data [38] and co-chromatography with the corresponding reference substances.

### 2.3. Ames Test

During the procedure, no precipitation of all tested concentrations of red and white AoBTHPs was observed in the culture plates in the whole battery of strains. The main results of the Ames test are presented in Table 3.

None of the tested concentrations of red and white AoBTHPs (250, 625, 1250, 2500, 3750, and 5000 μg/plate) induced an increase in the number of revertant colonies in bacterial strains TA98, TA 100, TA 102, TA 1335, and TA1537. In contrast, a clear increment in the amount of spontaneous revertants of the bacterial strains was found in all positive controls, compared with the negative control.

Moreover, a decrease in the background lawn of the plates was not observed in any tested concentrations of red and white AoBTHPs compared to the negative control.

### 2.4. Antioxidant Activity

A standard calibration curve was constructed for each spectrophotometric assay using ascorbic acid. The equations obtained were y = −0.0016x + 0.4024 and coefficient of determination (R^2^) = 0.994 for the DPPH assay; y = 0.0032x + 0.0452 and R^2^ = 0.993 for the FRAP assay; and y = 0.004x + 0.0009 and R^2^ = 0.999 for the TAC assay using the phosphomolybdenum method, where y is the absorbance, and x is the mg AAE/g AoBTHP AAE per µg of AoBTHP.

The spectrophotometric quantification of the in vitro antioxidant activity on red and white AoBTHPs is presented in Table 4.

The DPPH assay, FRAP assay, and TAC using the phosphomolybdenum method showed that red and white Portuguese AoBTHPs have similar antioxidant activities, with slight differences in each, without statistical significance (*p* < 0.05).

**Figure 2:**
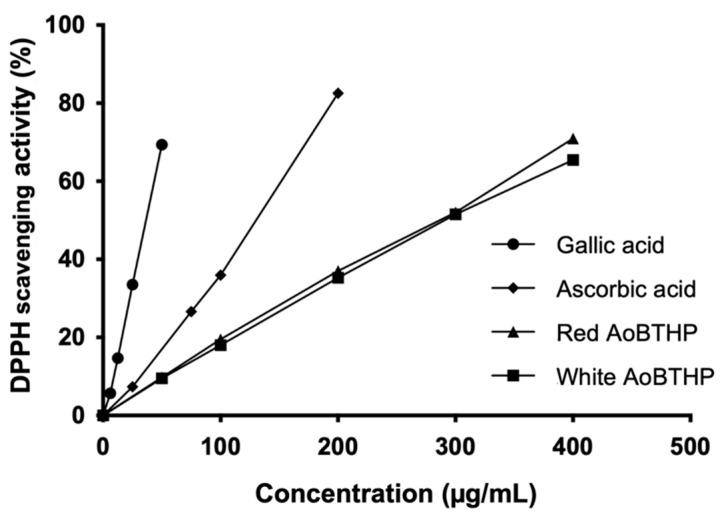
DPPH free radical scavenging activity.

The data show that both AoBTHPs exhibited concentration-dependent DPPH radical scavenging activity (Figure 2).

The half-maximal effective concentration (EC_50_) values calculated by the DPPH assay for the red and white AoBTHPs (280.7 ± 6.7 μg/mL and 296.8 ± 3.1 μg/mL, respectively) were significantly higher (*p* < 0.0001) compared to ascorbic acid (125.6 ± 3.9 μg/mL) and gallic acid (37.1 ± 0.6 μg/mL).

### 2.5. Anti-Inflammatory Activity

**Figure 3:**
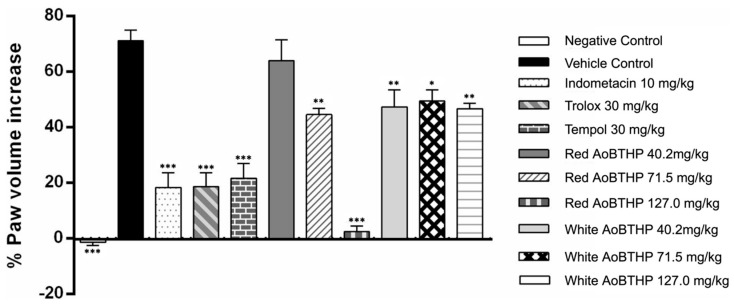
Evaluation of the anti-inflammatory activity of red and white AoBTHPs. *** *p* < 0.0001 versus vehicle control group; ** *p* < 0.001 versus vehicle control group; * *p* < 0.01 versus vehicle control group.

The effect of red and white AoBTHPs on carrageenan-induced rat paw edema was examined 6 h after carrageenan administration to analyze the percentage increase in paw volume relative to basal values and compared to control groups. These results are presented in Figure 3.

All groups except the negative control (the only group that did not receive carrageenan saline solution) showed that the volume of the paw volume at the end of the 6 h was higher than the basal paw volume.

The group that received the saline solution (vehicle control) showed a significative increment in carrageenan-induced paw edema (*p* < 0.0001) compared to the negative control (group showing no induced paw edema), validating the assay.

A significant antiedema effect of red Portuguese AoB THP 71.5 and 127.0 mg/kg (*p* < 0.001 and *p* < 0.0001, respectively) and white AoB THP 40.2, 71.5 and 127.0 mg/kg (*p* < 0.001, *p* < 0.01 and *p* < 0.001, respectively) was observed at the end of 6 h compared to vehicle control since the percentage increase in paw volume from baseline was significantly less.

The animals pre-treated with indomethacin 10 mg/kg, Trolox 30 mg/kg, and Tempol 30 mg/kg (positive control groups) also showed a significant reduction in carrageenan-induced paw edema compared to the vehicle control.

The red AoBTHP 127 mg/kg pre-treated group showed a minimal increase in paw volume compared to the basal value (2.46 ± 1.98%). This rat paw volume increment is smaller than that obtained with indomethacin 10 mg/kg (18.4 ± 5.2%), Trolox 30 mg/kg (18.6 ± 5.1%), and Tempol 30 mg/kg (21.7 ± 5.3%). It corresponds to an important inhibition of carrageenan-induced paw edema.

## 3. Discussion

The development of new drugs can be done by chemical synthesis or from natural resources (e.g., plants, fungi, or bacteria), isolating compounds with pharmacological activity, or using standardized extracts from these resources [39]. The use of medicinal plants to achieve health benefits is widely accepted and has led to the use of multiple effective drugs in therapy [40].

The current regulatory framework of herbal substances/preparations requires the assurance of an adequate safety profile [41]. Non-clinical safety is a basic requirement for applications for marketing authorization and simplified registration of herbal medicinal products [42]. This should include a comprehensive assessment of the genotoxic potential using in vitro and in vivo models of pro- and eukaryotic systems with and without metabolic activation [43].

In this work, we performed the Ames test, one of the most applied tests in toxicology, to determine the mutagenic potential of different substances [44]. This is a bacterial reverse gene mutation short-term test for the identification of carcinogens using mutagenicity in a set of different *Salmonella typhimurium* strains as an endpoint [45,46]. At tested concentrations (250–5000 μg of extracts/plate) of red and white AoBTHPs, no mutagenicity or evidence of cytotoxicity was observed, as neither an increment in the number of spontaneous revertants per plaque (the number of revertants was not significantly increased to at least 2-fold the negative control for TA98, TA100, and TA102, and 3-fold the negative control for TA1535 and TA1537) nor a decrease in the background lawn of the plates at any tested bacterial strain compared to the negative control [47]. Additionally, no dose–response relationship was observed between the tested concentrations of AoBTHPs. Therefore, according to the guidelines of ICH, OECD, and European Medicines Agency, under the conditions of this study and using plate incorporation without metabolic activation, the results obtained with red and white Portuguese AoBTHPs were unequivocally negative [43,48,49,50].

A review of the published research showed that these findings are consonant with the available data from the in vitro genotoxicity assessment of methanolic and ethanolic AoB extracts, which revealed no genotoxic effect on Chinese hamster lung fibroblasts (V79 cells) [51,52] and no cytotoxic activity in murine fibroblasts (L929) [53], respectively.

In this work, we did not perform the Ames test with metabolic activation. However, red and white Portuguese AoBTHPs were previously examined for their genotoxicity potential in vivo, so the pharmacokinetics of the substances were taken into account, and the absence of significant genotoxicity risk was demonstrated [23,49]. In addition, the main components of the formulations (gallic and protocatechuic acids) do not exhibit any direct or indirect genotoxicity [54,55]. Therefore, no further genotoxicity testing on red and white AoBTHPs is required to ensure their pre-clinical safety, and these data fulfill the genotoxicity testing requirements for inclusion of an herbal substance or preparation in the European Community list of herbal substances, preparations, and combinations thereof for use in traditional herbal medicinal products [43].

In the first LC-UV/DAD fingerprint, we detected the presence of gallic, protocatechuic, and ellagic acids in red and white Portuguese AoBTHPs [33,34]. Now, we have updated these data using a LC-UV/DAD-ESI-MS/MS analysis which has enabled a more exhaustive study of the formulations. The presence of gallic, protocatechuic, and ellagic acids was confirmed, and catechin, epicatechin, and epigallocatechin gallate were identified in red and white Portuguese AoBTHPs.

Previously, de Araújo Vilar et al. (2016) identified by LC-UV/DAD gallic acid, catechin, and epicatechin in the ethyl acetate phase of an acetone extract of AoB and their corresponding concentrations were found to be 397.61 μg/mL, 13.78 μg/mL, and 23.24 μg/mL [26]. Other authors, also using LC-UV/DAD, showed the presence of gallic acid, catechin, ellagic acid, and epicatechin in a methanolic extract of AoB and its ethanolic and ethyl acetate fractions [56]. Gallic acid was also identified in ethanolic AoB extracts using ultrahigh-performance liquid chromatography coupled with diode array detection and quadrupole time-of-flight mass spectrometry [38]. Nevertheless, we must note that none of these extracts are aqueous like the Portuguese AoBTHPs and that the solubility of the compounds varies depending on the solvent and the extraction technique. In addition, water-based procedures benefit from the use of a cheap, abundant, and health-harmless solvent [57].

In this research, we evaluated the antioxidant activities of red and white AoBTHPs, as these THPs have a high content of phenolic compounds, which are recognized as strong antioxidants [23,58].

Antioxidants play an important role in neutralizing free radicals in biological cells, reducing and preventing the development of oxidative stress [59]. The individual antioxidant activity of distinct compounds and their additive, synergistic, or antagonistic interactions can be integrated and ideally evaluated by different analytical methods, considering that the antioxidants are unequally reactive in all these tests [60]. The in vitro methods are cheap and high-throughput tools that are widely applied to discover new antioxidant substances [61]. So, in our research, we selected three different in vitro methods to characterize the potential antioxidant activities of red and white AoBTHPs and mitigate the inherent chemical limitations of each method. First, the DPPH test was performed, which relies on the antioxidants’ electron donation to neutralize the DPPH radical, causing a color change from deep purple to pale yellow [62]. The second was the FRAP assay, which is based on the reduction of ferric ions (Fe^3+^)-ligand to a ferrous complex (Fe^2+^) in the presence of an acidic medium and subsequent formation of an intense blue [60]. The last was TAC by the phosphomolybdenum method, which involves the reduction of Mo^6+^ to Mo^5+^ by the antioxidants and the subsequent formation of a phosphate Mo^5+^ complex under acidic conditions, resulting in the generation of a green or greenish-blue color [63].

Red and white AoBTHPs showed similar antioxidant activity in all three assays, but the antioxidant activity of ascorbic acid, an important physiological antioxidant [64], was comparatively more effective than that of AoBTHPs. As previously reported, red and white Portuguese AoBTHPs exhibited a concentration-dependent free radical scavenging activity using the DPPH assay [23]. This data was confirmed and complemented by the results obtained in the two complementary assays performed. Note that in the DPPH assay, gallic acid, a phenolic acid with high free radical scavenging activity [65] and the main compound of AoBTHPs, exhibited a lower EC_50_ value than ascorbic acid and consequently revealed the strongest free radical scavenging activity.

These data are consistent with previous studies conducted by other authors who had shown that different types of AoB extracts have antioxidant properties. Chaves et al. (2009) reported an AoB ethanolic extract concentration-dependent radical scavenging activity by DPPH assay (from 25 up to 100 μg/mL) [66]. Other studies performed with AoB ethanolic extracts, also using the DPPH assay, showed EC_50_ of 235.30 μg/mL [67], 1.12 μg/mL (a lower value than quercetin used as standard) [68], and 32.86 ± 3.05 μg/mL (ascorbic acid EC_50_ of 8.36 ± 6.63 μg/mL) [56]. Additionally, an AoB methanolic extract showed an EC_50_ of 42.44 ± 0.16 μg/mL (a lower value than the standard ascorbic acid, EC_50_ of 121.7 ± 0.04 μg/mL) [69] and AoB ethyl acetate, aqueous and methanolic extracts exhibited antioxidant activity by TAC and DPPH methods [70]. In this last study, the aqueous extract showed the lowest antioxidant activity in both assays, contrasting with the ethyl acetate extract, which revealed the highest activity.

To the best of our knowledge, this is the first report on the anti-inflammatory properties of red and white Portuguese AoBTHPs. To evaluate the anti-inflammatory activity of AoBTHPs, we selected the carrageenan-induced paw edema model in the rat, which is commonly used to study acute local inflammation and to search for and develop new anti-inflammatory drugs since edema is precisely one of the cardinal signs of inflammation [71,72,73] and three positive controls were used, namely indomethacin (a nonsteroidal anti-inflammatory drug) [74], Trolox (a water-soluble analog of vitamin E with potent antioxidant and anti-inflammatory effects) [75,76] and Tempol (a redox cycling nitroxide that has shown antioxidant, anti-inflammatory, anti-apoptotic and immunomodulatory activities) [77]. Analysis of the results obtained revealed that the two highest doses (71.5 and 127.0 mg/kg) of red AoB THP and all tested doses of white AoBTHP (40.2, 71.5, and 127.0 mg/kg) were effective in reducing the hind paw volume. The red AoBTHP 127 mg/kg produced a higher reduction in carrageenan-induced paw edema than indomethacin, Trolox, and Tempol, underscoring the potential anti-inflammatory activity of this herbal preparation.

Our results agree with those obtained by others using various samples and extracts of *A. occidentale* bark and different models of anti-inflammatory activity [26,27,28,29,30]. Worthy of highlighting, the AoB aqueous extract tested by Thomas et al. (2015), using the carrageenan-induced rat paw edema model at specific doses of 100, 200, and 400 mg/kg p.o., led to a reduction in rat paw edema of 45.03%, 48.17%, and 52.88%, respectively [29].

Anti-inflammatory and antioxidant activities are very closely related and often difficult to dissociate, since antioxidant mechanisms will lead to decreased inflammation processes [78].

The antioxidant properties of gallic and protocatechuic acids, the two main red and white Portuguese AoBTHP constituents we identified, have already been reported by other authors, and some details are known regarding the anti-inflammatory properties of these compounds. Gallic acid has been described as a pro-oxidant and antioxidant agent, and its therapeutic effects are mainly attributed to these properties, which include modulation of various signaling pathways by a variety of inflammatory cytokines and enzymatic and non-enzymatic antioxidants [65]. This compound also decreases the inflammatory response by reducing the release of inflammatory cytokines, chemokines, adhesion molecules, and cell infiltration through the MAPK and NF-κB signaling pathways [79]. Ben Saad et al. (2017) reported that gallic acid inhibited the production of nitric oxide (NO), PGE2, and interleukin 6 (IL-6) in LPS–induced RAW267.4 macrophages [80]. Other authors have found that the anti-inflammatory effect of gallic acid may be due to suppression of p65-NF-*κ*B and activation of IL-6/p-signal transducer and activator of transcription (STAT)3Y705 [81]. Relative to the anti-inflammatory properties of protocatechuic acid, Son et al. (2018) concluded that this compound may have potential benefits against LPS-induced excessive ROS formation and cell senescence [82]. In a streptozotocin-induced diabetic rat model, the protocatechuic acid incremented the antioxidant status, inhibiting lipid peroxidation, and suppressed pro-inflammatory biomarkers (myeloperoxidase activity, NO, and tumor necrosis factor-alpha (TNF-*α*) levels [83].

Since phenolic compounds are the major chemical class identified in red and white AoBTHPs and gallic and protocatechuic acids are recognized anti-inflammatory and antioxidant natural products, they may be involved in the anti-inflammatory and free radical scavenging and reducing activities of red and white AoBTHPs. However, the antioxidant and anti-inflammatory effects of red and white AoBTHPs may be due not only to the main components’ individual activity but also to synergisms between them. Therefore, it is essential to evaluate the formulations as a whole and not only their individual components.

Inflammatory diseases are considered the main cause of global morbidity [2], with oxidative stress playing a central role in the genesis of these conditions, which, as mentioned above, include diabetes mellitus, rheumatoid arthritis, and cancer [3,7]. There is an urgent need to find new therapeutic alternatives to treat and slow the progression of these diseases, which could involve exploring standardized herbal formulations [33] with recognized antioxidant activity, such as red and white Portuguese AoBTHPs that have proven to be safe at preclinical levels [23] and possess in vitro antioxidant, in vivo anti-inflammatory, in vitro and in vivo antidiabetic activities [34].

## 4. Materials and Methods

### 4.1. Chemicals and Reagents

Acetic acid glacial 99–100% and sulfuric acid were obtained from Chem-Lab^®^ (Zedelgem, Belgium). Acetonitrile was purchased from Honeywell Riedel-de Haën^TM^ (Seelze, Germany). Ammonium molybdate, hydrochloric acid fuming 37%, iron (III) chloride hexahydrate, L-histidine monohydrochloride monohydrate, sodium acetate trihydrate, and sodium dihydrogen phosphate dihydrate were obtained from Merck (Darmstadt, Germany). Ammonium sodium phosphate dibasic tetrahydrate, di-Potassium hydrogen phosphate anhydrous, and sodium chloride were obtained from Fluka (Seelze, Germany). Ascorbic acid, carrageenan, (±)-catechin hydrate, D-biotin, dimethyl sulfoxide (DMSO), ellagic acid, (−)-epicatechin, (−)-epigallocatechin gallate, indomethacin, protocatechuic acid, tert-butyl hydroperoxide, Tempol, Trolox, DPPH, 2-nitrofluorene, and 2,4,6-tris(2-pyridyl)-s-triazine (TPTZ) were purchased from Sigma-Aldrich^®^ (St. Louis, MO, USA). Bacto™ agar was obtained from Becton, Dickinson and Company (Sparks, MD, USA). Citric acid monohydrate, disodium hydrogen phosphate dihydrate, and sodium dihydrogen phosphate monohydrate were obtained from Panreac (Barcelona, Spain). Magnesium sulfate heptahydrate was obtained from (LabChem Inc., Zelienople, PA, USA). Methanol was obtained from Fisher Chemicals^®^ (Leicestershire, UK). Nutrient broth n° 2 was obtained from Oxoid (Basingstoke, UK). Sodium azide was obtained from J.T. Baker Chemical Company (Phillipsburg, NJ, USA). Sodium chloride 0.9% was acquired from B. Braun Medical, Lda. (Queluz, Portugal). All chemicals used were of analytical grade.

### 4.2. Plant Material

Fragments of red and white AoB types were gathered during fructification and identified in Guinea-Bissau by Professor Luís Catarino from the Department of Plant Biology, Faculty of Sciences of the Universidade de Lisboa. Corresponding voucher specimens were deposited in the LISC-Herbarium collection, Instituto de Investigação Científica Tropical (voucher numbers: red AoB gathered at Paiai, 11.836° N; 14.421° W: LC 1922 LC and white AoB gathered at Dulombi, 11.858° N; 14.503° W: LC1924 CJ). All samples were dried at room temperature away from direct light and stored in the Laboratory of Pharmacognosy, Faculty of Pharmacy of the Universidade de Lisboa.

Additionally, the plant name was checked in the online flora “The World Flora Online” [22].

### 4.3. Extract Preparation

Plant material was manually shredded into 1–2 cm fragments and homogenized as specified in European Pharmacopoeia 11.0 [84]. Subsequently, the extraction procedure was carried out according to the traditional method: Portuguese AoBTHPs were prepared extemporaneously by water maceration of the dried AoB (1:7 *w*/*v*) for 48 h at a controlled temperature (2–8 °C).

Then the extracts were filtered with cotton tissue (according to the traditional method of producing THP) and used in the in vivo anti-inflammatory activity test. Additionally, for the other assays, 20 mL aliquots of these filtered extracts were frozen at −20 °C and then lyophilized at −56 °C and kept in the freezer at −20 °C until their use.

### 4.4. LC-UV/DAD-ESI-MS/MS Phytochemical Profile

To perform the analysis, the red and white AoB samples were previously solubilized in acetonitrile (5 mg/mL) and filtered through a polytetrafluoroethylene syringe filter (0.2 μm).

Samples were injected in a volume of 10 μL into a LiChrospher^®^ 100 RP-18 end-capped particle size 5 μm, 100 Å, LiChroCART^®^ 250 × 4 mm (Merck, Darmstadt, Germany) and separated by LC Waters Alliance 2695 coupled to a Waters 2996 Photodiode Array Detector (PDA) (Waters Corporation, Milford, MA, USA). A MicroMass Quattromicro^®^ API triple quadrupole Mass Spectrometer (Waters^®^, Wexford, Ireland) was used to perform mass spectroscopy.

The LC-UV/DAD-ESI-MS/MS phytochemical profile was established according to the method described by Encarnação et al. (2022) with some modifications [34].

The mobile phase consisted of water/0.5% formic acid (solvent A) and acetonitrile (solvent B). Chromatographic separation was performed using a gradient elution of 5–17% B in 0 to 26 min, 17–33% B in 26 to 90 min, 33–40% B in 90 to 92 min, and 40–75% B in 92 to 101 min at a flow rate of 0.3 mL/min. The column was then washed and reconditioned. The temperature of the column thermostat was 25 °C. Data was registered and analyzed using Waters Millennium^®^32 Chromatography Manager (Waters Corporation, Milford, MA, USA). Chromatograms were monitored and registered on Maxplot (210–600 nm).

The compounds were ionized by an electrospray source in negative mode (ESI^−^), at 20 V con voltages. Data processing was performed with Waters MassLynx™ Software Version 4.1.

### 4.5. Ames Test

The in vitro genotoxic potential was evaluated using the Ames test protocol described by Maron and Ames (1983) [85] and according to current international requirements [48,50].

The test was performed using the direct plate incorporation method without metabolic activation with five tester strains of *Salmonella enterica* serovar Typhimurium (TA1537, TA1535, TA102, TA100, and TA98) [86], which were kindly provided by the Genetics Department of the Nova Medical School of the New University of Lisbon (Lisbon, Portugal). The *Salmonella enterica* strains were inoculated into an end-capped nutrient broth and incubated at 37 °C for 12–16 h and 210 rpm in the dark in an orbital incubator. They were then kept at 4 °C until use.

An aliquot of red or white Portuguese AoBTHP was diluted with 10% DMSO until the final volume was 200 µL, corresponding to doses of 250, 625, 1250, 2500, 3750, and 5000 μg/plate (the maximum dose level recommended by the Organization for Economic Co-operation and Development (OECD) guideline for testing chemicals) [50]. Then, 500 µL of sodium phosphate buffer (0.1 M, pH 7.4) and 100 µL of the bacterial culture were added to the extract. Finally, this mixture was mixed with 2 mL of molten Top-agar (melted at 100 °C and cooled to 45 °C), containing biotin and a trace of histidine, and plated in glucose minimal agar. After incubation at 37 °C for 48 h, the manual counting of His^+^ revertant colonies was performed for each extract/control plate, and the background lawn was examined for signs of toxicity or compound precipitation.

DMSO was used as a negative control and the solutions of 2-nitrofluorene (5 μg/plate), sodium azide (1.5 μg/plate), tert-butyl-hydroperoxide (50 μg/plate), and 9-aminoacridine (100 μg/plate) were used as positive controls for TA98, TA100 and TA1535, TA102, and TA1537 strains, respectively.

All tests were performed in triplicate, and results are presented as the mean number of revertant colonies per plate ± standard deviation (SD).

### 4.6. Antioxidant Activity Evaluation

The antioxidant properties of red and white Portuguese AoBTHPs were evaluated using three different spectrophotometric methods: DPPH method, FRAP assay, and TAC using the phosphomolybdenum method.

All values were determined in 3 sets of experiments and evaluated in triplicate using a Hitachi U-2000 UV-Vis spectrophotometer (Tokyo, Japan). The results are presented as mean ± SD.

#### 4.6.1. DPPH Assay

The activity as a free radical scavenger was assessed using the DPPH assay described by Silva et al. (2006) [87]. The assay was performed by adding 3.9 mL of DPPH solution (6 × 10^−5^ M in methanol) to 0.1 mL of extract/water/standard. After incubation at room temperature for 30 min, the absorbance of the solutions was measured spectrophotometrically at 517 nm against the blank. Increasing ascorbic acid concentrations (25.0–200.0 μg/mL) were used to obtain a standard curve. Results are expressed in milligrams of ascorbic acid equivalents (AAE) *per* gram of AoBTHP (mg AAE/g AoBTHP). Additionally, the percentage of DPPH free radical scavenging activity was calculated using the following formula: % scavenging = [control absorbance − absorbance of the test sample/control absorbance] × 100. Results are reported as the sample efficient concentration to reduce the initial DPPH concentration by 50% − EC_50_.

#### 4.6.2. Ferric Reducing Antioxidant Power Assay

The antioxidant activity was also evaluated according to the FRAP assay protocol described by Benzie and Strain (1996) with some modifications [88]. The FRAP reagent contained 10 mM TPTZ solution in 40 mM hydrochloric acid, 20 mM iron (III) chloride hexahydrate, and acetate buffer (300 Mm, pH = 3.6) (1:1:10, *v*/*v*/*v*). One hundred microliters of extract (50.0–400.0 μg/mL)/water/standard were added to 3 mL of FRAP reagent, and absorbance was measured at 593 nm with Hitachi U-2000 spectrophotometer (Tokyo, Japan) after incubation at room temperature for 4 min, using the FRAP reagent as blank. The reference standard was ascorbic acid (25.0–200.0 μg/mL). Results are expressed as mg AAE/g AoBTHP.

#### 4.6.3. Total Antioxidant Capacity Assay

The TAC assay was carried out using the phosphomolybdenum method described by Prieto et al. (1999) [89]. Two hundred microliters of extract (50.0–400.0 μg/mL)/water/standard were mixed with 2 mL of the reagent solution containing 0.6 M sulfuric acid, 28 mM sodium phosphate, and 4 mM ammonium molybdate. The vials were capped and incubated in a water bath at 95 °C for 90 min. After the samples had cooled to room temperature, the absorbance was measured spectrophotometrically at 695 nm against the blank. Increasing ascorbic acid concentrations (25.0–200.0 μg/mL) were used to obtain a standard curve. Results are expressed as milligrams of AAE per gram of AoBTHP (mg AAE/g AoBTHP).

### 4.7. Anti-Inflammatory Activity

#### 4.7.1. Animals

Sixty-seven male Wistar rats weighing 150.8 ± 3.2 g were purchased from Harlan Laboratories Inc. (Barcelona, Spain). The rats were housed in groups of five or six animals per cage in the Animal House of the Faculty of Pharmacy of the University of Lisbon under controlled environmental conditions with a 12–12-h light-dark cycle, a temperature of 22 ± 2 °C and a relative humidity of 55 ± 10%. All animals received water and standard laboratory rat chow ad libitum. The animals were acclimatized to laboratory conditions three weeks before the start of the study.

#### 4.7.2. Experimental Protocol

The anti-inflammatory activity was evaluated according to the protocol of the carrageenan-induced paw edema in rats described by Rocha et al. (2015) [90]. This experimental protocol was approved in February 2016 by the Ethics Committee for Animal Experiments (CEEA) of the Faculty of Pharmacy of the Universidade de Lisboa (protocol CEEE-002/16). The experiments were performed in agreement with European and Portuguese ethical requirements [91,92].

Sixty-seven male Wistar rats were randomized by weight and divided into 11 groups. The water, drugs or extracts were administered by oral gavage 10 mL/kg body weight (BW) [93] as described: (i) Group 1 (negative control): animals received vehicle (water) (n = 5); (ii) Group 2 (vehicle control group—carrageenan): animals received vehicle (water) (n = 11); (iii) Group 3 (indomethacin group): animals pre-treated with indomethacin (10 mg/kg) (n = 5); (iv) Group 4 (Trolox group): animals pre-treated with Trolox (30 mg/kg) (n = 5); (v) Group 5 (Tempol group): animals pre-treated with Tempol (30 mg/kg) (n = 5); (vi) Groups 6–8 (red AoBTHP groups): animals pre-treated with red AoBTHP 40.2, 71.5 or 127.0 mg/kg (n = 6); (vii) Groups 9–11 (white AoBTHP groups): animals pre-treated with white AoBTHP 40.2, 71.5 or 127.0 mg/kg (n = 6).

After 1 h, paw edema was induced in all rats by a single subplantar injection of 0.1 mL of 1% carrageenan saline solution in the left hind paw (except in the negative control group that received a subplantar injection of 0.1 mL of sterile saline).

Paw volume was measured immediately after the injection of carrageenan (basal volume), and 6 h later using a Digital Plethysmometer LE7500 (Letica Scientific Instruments—Reagente 5, Porto, Portugal). Paw edema is presented as mean ± standard error of the mean (SEM) and expressed as a percentage of the increase in paw volume 6 h after carrageenan injection relative to basal values according to the equation: % = [(V_6_ − V_0_)/V_0_] × 100, where V_0_ = paw volume measured immediately after carrageenan injection, and V = paw volume measured 6 h after carrageenan injection.

#### 4.7.3. Rationale for Dose Selection and Route of Administration

The selected THP doses (40.2, 71.5, and 127.0 mg/kg BW/day) were previously tested in a study developed by our team in db/db mice (type 2 diabetes mouse model) that evaluated the hypoglycemic activity of Portuguese AoBTHP in diabetes [34]. In our study, AoBTHP caused a dose-dependent decrease in fasting blood glucose. All samples were administered orally by gavage, the most common route of drug administration in humans.

### 4.8. Statistical Analysis

Data were analyzed using Microsoft Excel version 16.49 (Microsoft Corporation, Redmond, WA, USA) and GraphPad Prism version 5.0 for Windows (GraphPad Software Inc., San Diego, CA, USA).

EC_50_ values were calculated by linear regression analysis. The values are presented as mean ± SD or mean ± SEM and were assessed using *t*-test or one-way analysis of variance, followed by Bonferroni’s multiple comparison test. Differences were considered statistically significant if the *p*-value was less than 0.05.

## 5. Conclusions

This work assessed the in vivo anti-inflammatory and in vitro antioxidant activities of the red and white Portuguese AoBTHPs as well as their in vitro genotoxic potential. LC-UV/DAD-ESI-MS/MS phytochemical profiles were also established, and major compounds identified.

The carrageenan-induced paw edema rat model showed an effective anti-inflammatory activity of both AoBTHPs and the highest concentration of the red AoBTHP (127.0 mg/kg) was much more effective than clinically relevant substances used as controls (indomethacin 10 mg/kg, Trolox 30 mg/kg and Tempol 30 mg/kg). The exhibited anti-inflammatory activity and the antioxidant potential, aligned with lack of genotoxicity in accordance with the requirements of the ICH guideline, support the concrete validation of red and white AoB and their THPs as anti-inflammatory agents and contribute for the possible development of promising new therapeutic options to treat inflammatory conditions.

In future research, enzymatic models should be employed to clarify the mechanisms of action underlying the anti-inflammatory activity, and data from in vitro biological assays and/or in vivo assessment should be added to the analysis of the antioxidant activity of whole extracts and isolated marker active compounds to allow for a more detailed structure-activity correlation and based on these, the establishment of more assertive parameters for the standardization of AoBTHPs.

## Figures and Tables

**Table 1:** In vivo studies on the anti-inflammatory effects of *A. occidentale* stem bark extracts.

Type of Extraction	Extract Dose and Route of Administration	Duration of Study	Positive Control	Model	Results	Ref.
Acetone	0.1, 0.3 and 1 g/kg p.o.	5 h	Indomethacin 10 mg/kg p.o.	Acetic acid-induced abdominal writhing	Dose-related inhibition of acetic acid-induced abdominal writhing (18.9–62.9%)	[30]
Acetone	1 g/kg p.o.	5 h	Indomethacin 10 mg/kg p.o.	Croton oil-induced ear edema	Inhibition of the edematogenic response after topical application of croton oil (56.8%)	[30]
Acetone	0.1, 0.3 and 1 g/kg p.o.	5 h	Dexamethasone 2 mg/kg s.c.	Carrageenan-induced peritonitis	Dose-related reduction of leukocyte migration (24.8–49.6%)	[30]
Acetone and ethyl acetate	12.5, 25, 50 and 100 mg/k p.o.	7 h	Indomethacin 10 mg/kg i.p.	Carrageenan, Bradykinin, and Prostaglandin-induced mice paw edema	Dose- and time-dependent effects in reducing paw edema in mice. Significant antiedema effect after PGE2 and bradykinin challenge.	[26]
Acetone and ethyl acetate	50 and 100 mg/kg p.o.	11 h	Indomethacin 10 mg/kg i.p.	Carrageenan-induced peritonitis	Inhibition of carrageenan-induced leukocyte number in the peritoneal cavity at 4 h (57%).	[26]
Aqueous	800 mg/kg p.o.	n/a	Diclofenac 100 mg/kg p.o.	Egg albumin-induced rat paw edema	Time-related reduction of the rat’s hind paw	[27]
Aqueous	100, 200, 400 mg/kg p.o.	7 h	Indomethacin 10 mg/kg p.o.	Carrageenan-induced rat paw edema	Rat paw edema reduction (45.03, 48.17 and 52.88%)	[29]
Ethanol	100, 200, 400 mg/kg p.o.	7 h	Indomethacin 10 mg/kg p.o.	Carrageenan-induced rat paw edema	Rat paw edema reduction (60.73, 68.32 and 70.68%)	[29]
Methanol	25, 50, 100 and 200 mg/kg i.p.	8 h 30 min	Entoxifylline 100 mg/kg, i.p.; L-NAME 5 mg/kg	LPS-induced septic shock in Swiss mice	Dose-dependent effect in mortality reduction (83–0% of mortality)	[28]
Methanol	25, 50, 100 and 200 mg/kg i.p.	8 h 30 min	Entoxifylline 100 mg/kg, i.p.; L-NAME 5 mg/kg	LPS-induced plasma leakage in Swiss mice skin	Dose-dependent inhibition of dye leakage	[28]

i.p.—intraperitoneal; i.v.—intravenous; LPS—Lipopolysaccharide; n/a—not available; PGE2—prostaglandin E2; p.o.—*per os*; Ref.—Reference; s.c.—subcutaneous.

**Table 2:** LC-UV/DAD-ESI-MS/MS-based identification of red *A. occidentale* traditional herbal preparation marker compounds.

Peak N	R_t_ (min)	UV-Vis (λ_max_)	Molecular Formula	Molecular Weight ^a^	[M − H]^−^ (*m/z*)	MS/MS Fragmention (*m/z*)	Compound Name
1	16.21	272	C_7_H_6_O_5_	170.12	169	125	Gallic acid
2	24.71	260, 294	C_7_H_6_O_4_	154.12	153	109	Protocatechuic acid
3	32.32	237, 275	C_15_H_14_O_6_	290.27	289	245, 137, 125, 109	Catechin
4	37.87	237, 274	C_15_H_14_O_6_	290.27	289	245, 137, 125, 109	Epicatechin
5	38.66	275	C_22_H_18_O_11_	458.4	457	305, 288, 169, 125	Epigallocatechin gallate
6	51.79	253, 369	C_14_H_6_O_8_	302.19	301	283, 257, 229, 185	Ellagic acid

Peak N: Peak number; Rt: retention time; [M − H]^−^: negative mass electrospray ionization mode. ^a^ Retired from PubChem database [37].

**Table 3:** The plate incorporation test without metabolic activation.

Samples (µg/Plate)		Revertant Colonies per Plate (Mean ± SD)				
		TA98	TA100	TA102	TA1535	TA1537
Red AoB THP	5000	24.0 ± 2.65	135.3 ± 11.7	228.3 ± 6.4	15.3 ± 0.6	18.0 ± 2.0
	3750	21.0 ± 3.61	127.7 ± 15.3	243.3 ± 9.7	15.7 ± 2.5	15.0 ± 2.6
	2500	22.0 ± 1.73	171.0 ± 12.2	297.0 ± 51.2	13.0 ± 1.7	16.7 ± 1.5
	1250	23.7 ± 3.21	186.3 ± 8.1	221.7 ± 18.6	12.3 ± 5.0	14.7 ± 2.5
	625	24.7 ± 4.93	186.3 ± 11.0	280.0 ± 15.6	10.3 ± 1.5	15.0 ± 1.7
	250	23.7 ± 4.73	181.0 ± 17.7	305.0 ± 11.4	11.7 ± 2.3	14.7 ± 3.2
White AoB THP	5000	31.7 ± 1.53	114.3 ± 12.9	259.0 ± 5.6	13.7 ± 2.3	17.3 ± 0.6
	3750	27.3 ± 2.52	144.0 ± 5.3	258.7 ± 7.8	14.0 ± 1.0	16.0 ± 0.0
	2500	32.7 ± 0.58	133.3 ± 10.8	286.7 ± 34.3	12.0 ± 2.0	17.7 ± 3.1
	1250	31.0 ± 1.00	147.0 ± 13.7	296.0 ± 15.1	14.7 ± 2.3	15.0 ± 0.0
	625	30.7 ± 0.58	142.7 ± 11.6	291.0 ± 28.1	12.7 ± 3.1	14.7 ± 1.5
	250	28.3 ± 4.16	151.3 ± 22.2	286.7 ± 9.7	13.7 ± 3.1	14.3 ± 2.1
Negative Control		23.3 ± 0.58	166.7 ± 25.8	276.0 ± 14.1	14.3 ± 1.5	14.3 ± 2.3
Positive Control		487.7 ± 30.2 ^a^	1048.0 ± 43.2 ^b^	881.0 ± 26.2 ^c^	827.3 ± 13.1 ^b^	179.7 ± 19.4 ^d^

SD: Standard deviation; ^a^ 2-nitrofluorene (5 μg/plate); ^b^ sodium azide (1.5 μg/plate); ^c^ tert-butyl-hydroperoxide (50 μg/plate); ^d^ 9-aminoacridine (100 μg/plate).

**Table 4:** Spectrophotometric quantification of the antioxidant activity on *A. occidentale* bark extracts.

Antioxidant Activity Assay	AoBTHP	
	Red Type	White Type
DPPH (mg AAE/g AoBTHP)	508.95 ± 16.81	487.18 ± 14.33
FRAP (mg AAE/g AoBTHP)	398.95 ± 3.96	405.29 ± 5.60
TAC (mg AAE/g AoBTHP)	335.29 ± 23.48	346.17 ± 12.52

AAE: ascorbic acid equivalents; DPPH: 1,1-diphenyl-2-picrylhydrazyl; FRAP: Ferric Reducing Antioxidant Power; TAC: Total Antioxidant Capacity. There were no statistically significant differences between all groups (*p* < 0.05).

## Data Availability

Data is contained within the article.
